# New Susceptibility and Resistance HLA-DP Alleles to HBV-Related Diseases Identified by a Trans-Ethnic Association Study in Asia

**DOI:** 10.1371/journal.pone.0086449

**Published:** 2014-02-10

**Authors:** Nao Nishida, Hiromi Sawai, Koichi Kashiwase, Mutsuhiko Minami, Masaya Sugiyama, Wai-Kay Seto, Man-Fung Yuen, Nawarat Posuwan, Yong Poovorawan, Sang Hoon Ahn, Kwang-Hyub Han, Kentaro Matsuura, Yasuhito Tanaka, Masayuki Kurosaki, Yasuhiro Asahina, Namiki Izumi, Jong-Hon Kang, Shuhei Hige, Tatsuya Ide, Kazuhide Yamamoto, Isao Sakaida, Yoshikazu Murawaki, Yoshito Itoh, Akihiro Tamori, Etsuro Orito, Yoichi Hiasa, Masao Honda, Shuichi Kaneko, Eiji Mita, Kazuyuki Suzuki, Keisuke Hino, Eiji Tanaka, Satoshi Mochida, Masaaki Watanabe, Yuichiro Eguchi, Naohiko Masaki, Kazumoto Murata, Masaaki Korenaga, Yoriko Mawatari, Jun Ohashi, Minae Kawashima, Katsushi Tokunaga, Masashi Mizokami

**Affiliations:** 1 The Research Center for Hepatitis and Immunology, National Center for Global Health and Medicine, Ichikawa, Chiba, Japan; 2 Department of Human Genetics, Graduate School of Medicine, The University of Tokyo, Bunkyo-ku, Tokyo, Japan; 3 HLA Laboratory, Japanese Red Cross Kanto-Koshinetsu Block Blood Center, Koutou-ku, Tokyo, Japan; 4 Department of Medicine, Queen Mary Hospital, Hong Kong; 5 Faculty of Medicine, Chulalongkorn University, Bangkok, Thailand; 6 Department of Internal Medicine, Yonsei University College of Medicine, Seoul, South Korea; 7 Department of Virology & Liver Unit, Nagoya City University Graduate School of Medical Sciences, Nagoya, Aichi, Japan; 8 Division of Gastroenterology and Hepatology, Musashino Red Cross Hospital, Musashino, Tokyo, Japan; 9 Department of Liver Disease Control, Tokyo Medical and Dental University, Bunkyo-ku, Tokyo, Japan; 10 Department of Gastroenterology and Hepatology, Tokyo Medical and Dental University, Bunkyo-ku, Tokyo, Japan; 11 Department of Internal Medicine, Teine Keijinkai Hospital, Sapporo, Hokkaido, Japan; 12 Department of Internal Medicine, Hokkaido University Graduate School of Medicine, Sapporo, Hokkaido, Japan; 13 Division of Gastroenterology, Department of Medicine, Kurume University School of Medicine, Kurume, Fukuoka, Japan; 14 Department of Gastroenterology and Hepatology, Okayama University Graduate School of Medicine, Dentistry, and Pharmaceutical Sciences, Okayama, Okayama, Japan; 15 Gastroenterology and Hepatology, Yamaguchi University Graduate School of Medicine, Ube, Yamaguchi, Japan; 16 Faculty of Medicine, Tottori University, Tottori, Tottori, Japan; 17 Molecular Gastroenterology and Hepatology, Kyoto Prefectural University of Medicine, Kyoto, Kyoto, Japan; 18 Department of Hepatology, Osaka City University Graduate School of Medicine, Osaka, Osaka, Japan; 19 Department of Gastroenterology, Nagoya Daini Red Cross Hospital, Nagoya, Aichi, Japan; 20 Department of Gastroenterology and Metabology, Ehime University Graduate School of Medicine, Toon, Ehime, Japan; 21 Department of Gastroenterology, Kanazawa University Graduate School of Medicine, Kanazawa, Ishikawa, Japan; 22 Department of Gastroenterology and Hepatology, National Hospital Organization Osaka National Hospital, Osaka, Osaka, Japan; 23 Division of Gastroenterology and Hepatology, Department of Internal Medcine, Iwate Medical University, Morioka, Iwate, Japan; 24 Division of Hepatology and Pancreatology, Kawasaki Medical College, Kurashiki, Okayama, Japan; 25 Department of Medicine, Shinshu University School of Medicine, Matsumoto, Nagano, Japan; 26 Division of Gastroenterology and Hepatology, Saitama Medical University, Iruma, Saitama, Japan; 27 Department of Gastroenterology, Kitasato University School of Medicine, Sagamihara, Kanagawa, Japan; 28 Department of Internal Medicine, Saga Medical School, Saga, Saga, Japan; 29 Faculty of Medicine, University of Tsukuba, Tsukuba, Ibaraki, Japan; University of Pisa, Italy

## Abstract

Previous studies have revealed the association between SNPs located on human leukocyte antigen (*HLA*) class II genes, including *HLA-DP* and *HLA-DQ*, and chronic hepatitis B virus (HBV) infection, mainly in Asian populations. *HLA-DP* alleles or haplotypes associated with chronic HBV infection or disease progression have not been fully identified in Asian populations. We performed trans-ethnic association analyses of *HLA-DPA1*, *HLA-DPB1* alleles and haplotypes with hepatitis B virus infection and disease progression among Asian populations comprising Japanese, Korean, Hong Kong, and Thai subjects. To assess the association between *HLA-DP* and chronic HBV infection and disease progression, we conducted high-resolution (4-digit) *HLA-DPA1* and *HLA-DPB1* genotyping in a total of 3,167 samples, including HBV patients, HBV-resolved individuals and healthy controls. Trans-ethnic association analyses among Asian populations identified a new risk allele HLA-DPB1*09∶01 (P = 1.36×10^−6^; OR = 1.97; 95% CI, 1.50–2.59) and a new protective allele DPB1*02∶01 (P = 5.22×10^−6^; OR = 0.68; 95% CI, 0.58–0.81) to chronic HBV infection, in addition to the previously reported alleles. Moreover, DPB1*02∶01 was also associated with a decreased risk of disease progression in chronic HBV patients among Asian populations (P = 1.55×10^−7^; OR = 0.50; 95% CI, 0.39–0.65). Trans-ethnic association analyses identified Asian-specific associations of *HLA-DP* alleles and haplotypes with HBV infection or disease progression. The present findings will serve as a base for future functional studies of HLA-DP molecules in order to understand the pathogenesis of HBV infection and the development of hepatocellular carcinoma.

## Introduction

Hepatitis B virus (HBV) infection is a major global health problem, resulting in 0.5–1.0 million deaths per year [1]. The prevalence of chronic HBV infection varies. About 75% of the chronic carriers in the world live in Southeast Asia and East Pacific [2]. Due to the introduction of vaccination programs, the prevalence of HBV infection in many countries has gradually been decreasing with consequent decreases in HBV-related hepatocellular carcinoma (HCC) [3]. Although some HBV carriers spontaneously eliminate the virus, about 10–15% of carriers develop liver cirrhosis (LC), liver failure and HCC [4]. Moreover, the progression of liver disease was revealed to be associated with the presence of several distinct mutations in HBV infections [5]. Genetic variations in *STAT4* and *HLA-DQ* genes were recently identified as host genetic factors in a large-scale genome-wide association study (GWAS) for HBV-related HCC in China [6].

With regard to the genes associated with susceptibility to chronic HBV infection, *HLA-DP* and *HLA-DQ* genes were identified by GWAS in Japanese and Thai populations in 2009 [7] and 2011 [8], respectively. In addition, our previous GWAS confirmed and identified the association of SNP markers located on *HLA-DPA1* (rs3077) and *HLA-DPB1* (rs9277535) genes with susceptibility to chronic hepatitis B (CHB) and HBV clearance in Japanese and Korean subjects[9]. The significant associations of *HLA-DP* with CHB and HBV clearance have mainly been detected in Asian populations, such as Japanese [8], [9], Thai [7], Chinese [10]–[12], and Korean [9]. In 2012, the association between *HLA-DPA1* gene SNPs and persistent HBV infection was replicated in a Germany non-Asian population for the first time; however, this showed no association with HBV infection [13]. These results seem to be explained by the fact that allele frequencies of both rs3077 (0.155, 0.587 and 0.743 for C allele, on HapMap CEU, JPT, and YRI) and rs9277535 (0.261, 0.558 and 0.103 for G allele, on HapMap CEU, JPT, and YRI) are markedly different between populations. Moreover, the previous study showed that HBsAg seropositivity rates were higher in Thailand and China (5–12%) than in North America and Europe (0.2–0.5%) [2]. These results suggest that comparative analyses of *HLA-DP* alleles and haplotypes in Asian populations would clarify key host factors of the susceptible and protective *HLA-DP* alleles and haplotypes for CHB and HBV clearance. Here, we performed trans-ethnic analyses of *HLA-DP* alleles and haplotypes in Asian populations comprising Japanese, Korean, Hong Kong and Thai individuals. The findings from this study will serve as a base for future functional studies of HLA-DP molecules.

## Results

### Characteristics of studied subjects

The characteristics of a total of 3,167 samples, including Japanese, Korean, Hong Kong and Thai subjects, are shown in Table 1. Each population included three groups of HBV patients, resolved individuals and healthy controls. The clinical definitions of HBV patients and resolved individuals are summarized in Materials and Methods. Some of the Japanese and all of the Korean samples overlapped with the subjects in our previous study [9], [14].

**Table 1:** Number of individuals in this study.

Population	Japanese	Korean	Hong Kong	Thai
Total number of samples	1,291	586	661	629
HBV patients	489	340	281	390
IC	114	-	-	-
CH	147	175	187	198
AE	21	-	-	-
LC	38	-	-	-
HCC	169	165	94	192
Mean age (y)	57.1	44.7	57.9	52.0
(min-max)	(20–84)	(18–74)	(32–86)	(21–84)
Gender (M/F)	338/151	265/75	239/42	289/101
Resolved individuals*	335	106	190	113
HCV (−)	249	106	190	113
HCV (+)	86	-	-	-
Mean age (y)	59.7	43.1	40.0	48.2
(min-max)	(18–87)	(12–66)	(18–60)	(39–66)
Gender (M/F)	173/162	61/45	113/77	83/30
Healthy controls	467	140	190	126
Mean age (y)	39.0**	33.7	26.2	46.6
(min-max)	(23–64)	(1–59)	(16–60)	(38–79)
Gender (M/F)	370/97	67/73	87/103	73/53

Abbreviation: IC, Inactive Carrier; CH, Chronic Hepatitis; AE, Acute Exacerbation; LC, Liver Cirrhosis; HCC, Hepatocellular Carcinoma.

* Resolved individuals were HBsAg negative and HBcAb positive.

** 419 of 467 healthy controls were de-identified, without information on age.

We performed genotyping for *HLA-DPA1* and *HLA-DPB1* in all 3,167 samples, and a total of 2,895 samples were successfully genotyped. The characteristics of successfully genotyped samples are shown in Table S1.

### Association of *HLA-DPA1* and *HLA-DPB1* alleles in Asian populations

As for a general Asian population, including 464 Japanese, 140 Korean, 156 Hong Kong, and 122 Thai subjects, five *HLA-DPA1* alleles and twenty-four *HLA-DPB1* alleles were observed (Table S2). The frequencies of *HLA-DPA1* and *HLA-DPB1* alleles were similar between Japanese and Korean subjects. On the other hand, the number of alleles with frequencies of 1–2% was larger in Hong Kong and Thai populations, despite the small sample size. Although the frequencies of *HLA-DP* alleles varied in Asian populations, HLA-DPB1*05∶01 was the most prevalent with over 30% in all populations.

The associations of *HLA-DPA1* and *HLA-DPB1* alleles with chronic HBV infection (i.e., comparison between HBV patients and healthy controls) are shown in Table S2. To avoid false positives caused by multiple testing, the significance levels were corrected based on the numbers of *HLA-DPA1* and *HLA-DPB1* alleles in the focal population. Briefly, the significance level was set at 0.05/(# of observed alleles at each locus) in each population (see Materials and Methods). With regard to high-risk alleles of *HLA-DPA1*, the most prevalent allele HLA-DPA1*02∶02 was significantly associated with susceptibility to HBV infection in Japanese (P = 3.45×10^−4^; OR = 1.39; 95% CI, 1.16–1.68) and Korean subjects (P = 2.66×10^−5^; OR = 1.89; 95% CI, 1.39–2.58), whereas this association was not observed in Hong Kong or Thai subjects. The association of HLA-DPA1*02∶01 with susceptibility to HBV infection was significant only in Japanese (P = 2.61×10^−7^; OR = 1.88; 95% CI, 1.46–2.41). The significant association of HLA-DPA1*01∶03 with protection against HBV infection was commonly observed among four Asian populations (Table S2). The pooled OR and 95% CI were 0.51 and 0.41–0.63, respectively in a meta-analysis (P = 3.15×10^−10^) (Fig. S1A).

As shown in Table S2, *HLA-DPB1* shows higher degree of polymorphism than *HLA-DPA1*. The most common allele in Asian populations, HLA-DPB1*05∶01, was significantly associated with HBV susceptibility in both Japanese and Korean subjects. Although HLA-DPB1*05∶01 showed no significant association in the Hong Kong and Thai populations, the same direction of association (i.e., HBV susceptibility) was observed. Meta-analysis of the four populations revealed a significant association between HLA-DPB1*05∶01 and susceptibility to HBV infection (P = 1.51×10^−4^; OR = 1.45; 95% CI, 1.19–1.75) (Fig. S1B). The frequency of HLA-DPB1*09∶01 was significantly elevated in Japanese HBV patients (15.7%) as compared with healthy controls (8.7%) (P = 3.70×10^−6^; OR = 1.94; 95% CI, 1.45–2.62), and this association was most significant (i.e., the smallest P value) in the Japanese population. Because of lower allele frequencies of HLA-DPB1*09∶01 or lack of statistical power in the other populations, no significant associations were observed. A common allele in Thai subjects, HLA-DPB1*13∶01, was significantly associated with susceptibility to HBV infection (P = 2.49×10^−4^; OR = 2.17; 95% CI, 1.40–3.47) with the same direction of associations in Japanese and Hong Kong (OR = 1.52 and 1.40, respectively).

HLA-DPB1*04∶02 was identified as the most protective allele for HBV infection in Japanese (P = 1.59×10^−7^; OR = 0.37; 95% CI, 0.24–0.55) and Korean subjects (P = 1.27×10^−7^; OR = 0.19; 95% CI, 0.10–0.38). Both HLA-DPB1*02∶01 and HLA-DPB1*04∶01 were also significantly associated with protection in the Japanese population, and the former was significantly associated with protection in Hong Kong subjects (P = 9.17×10^−4^; OR = 0.49; 95% CI, 0.32–0.76). This common allele among four Asian populations, HLA-DPB1*02∶01, showed a significant association with protection against HBV infection (P = 5.22×10^−6^; OR = 0.68; 95% CI, 0.58–0.81) in a meta-analysis (Fig. S1B).

The frequencies of associated *HLA-DP* alleles in a comparison of HBV patients with healthy controls (Table S2) or with HBV-resolved individuals (Table S3) were similar in all four Asian populations. In the Japanese population, the associations of susceptible and protective *HLA-DPB1* alleles to chronic HBV infection seem weaker in the comparison of HBV patients with HBV-resolved individuals than in the comparison of HBV patients with healthy controls. Moreover, the results of association analyses showed no difference in the comparison of HBV patients with HBV-resolved individuals, including or excluding HCV positive individuals (Table S3). In contrast, the association became stronger in the comparison of HBV patients with HBV-resolved individuals among the Korean subjects. The protective allele HLA-DPB1*04∶01 was also identified to have a strong association with HBV clearance in Hong Kong subjects (Table S3). Moreover, in Hong Kong subjects, the HLA-DPB1*05∶01 associated with the risk for HBV infection showed lower frequency in HBV-resolved individuals (42.9%) than in the healthy controls (48.1%), which accounts for a strong association in the comparison of HBV patients with HBV-resolved individuals (P = 6.24×10^−3^; OR = 1.64; 95% CI, 1.14–2.36). Although the number of samples was insufficient, HLA-DP*100∶01 showed a significant association with protection against HBV infection in the Hong Kong population (P = 3.05×10^−6^; OR = 0.03; 95% CI, 0.0007–0.20).

As for disease progression in CHB patients among Asian populations, a protective effect of HLA-DPB1*02∶01 on disease progression was observed in the Japanese (P = 4.26×10^−5^; OR = 0.45; 95% CI, 0.30–0.67) and Korean populations (P = 8.74×10^−4^; OR = 0.47; 95% CI, 0.29–0.75) (Table S4). Multivariate logistic regression analysis adjusted for age and sex revealed that the number of DPB1*02∶01 alleles (i.e., 0, 1, or 2) was significantly associated with disease progression in CHB patients in Japanese (P = 1.77×10^−4^; OR = 0.47; 95% CI, 0.32–0.70) (Table 2). Moreover, protective effects of DPB1*02∶01 on disease progression in Asian populations (P = 1.55×10^−7^; OR = 0.50; 95% CI, 0.39–0.65) were detected in a multivariate logistic regression analysis adjusted for age, gender, and population (Table 2).

**Table 2:** Association of number of *DPB1* * *02∶01* alleles (i.e., 0, 1 or 2) with disease progression in CHB patients assessed by multivariate logistic regression analysis adjusted for age and sex.

Population	P value	OR (95% CI)
Japanese	0.000177	0.47 (0.32–0.70)
Korean	0.025358	0.55 (0.33–0.93)
Hong Kong	0.040842	0.46 (0.22–0.97)
Thai	0.087782	0.58 (0.31–1.08)
All*	1.55×10^−7^	0.50 (0.39–0.65)

*Population was adjusted using dummy variables.

### Associations of *DPA1-DPB1* haplotypes in Asian populations

The estimated frequencies of *HLA DPA1-DPB1* haplotypes are shown in Table S5. The most frequent haplotype among the four Asian populations was DPA1*02∶02-DPB1*05∶01. The number of haplotypes with low frequencies of 1–2% was 10 in both Japanese and Korean subjects, whereas more haplotypes appeared with frequencies of 1–2% in Hong Kong and Thai subjects. The associations of *DPA1-DPB1* haplotypes with HBV infection are shown in Table S5. In the Japanese population, DPA1*02∶01-DPB1*09∶01 showed the most significant association with susceptibility to HBV infection (P = 3.38×10^−6^; OR = 1.95; 95% CI, 1.46–2.64). The most common haplotype in the four Asian populations, DPA1*02∶02-DPB1*05∶01, was found to be significantly associated with susceptibility to HBV infection in the Japanese and Korean subjects (P = 7.40×10^−4^; OR = 1.37; 95% CI, 1.14–1.66 for Japanese, and P = 4.50×10^−6^; OR = 2.02; 95% CI, 1.48–2.78 for Korean). In the Thai subjects, HLA-DPB1*13∶01 was the most significant risk allele for HBV infection (Table S2); however, no significant associations were found for the three different haplotypes bearing HLA-DPB1*13∶01: DPA1*02∶01-DPB1*13∶01, DPA1*02∶02-DPB1*13∶01, and DPA1*04∶01-DPB1*13∶01,indicating that the association of HLA-DPB1*13∶01 with susceptibility to HBV infection did not result from a specific *DPA1-DPB1* haplotype or combination with a specific *DPA1* allele.

In the Japanese population, both haplotypes DPA1*01∶03-DPB1*04∶01 and DPA1*01∶03-DPB1*04∶02 showed significant associations with protection against HBV infection (P = 1.17×10^−5^; OR = 0.32; 95% CI, 0.18–0.56 for DPA1*01∶03-DPB1*04∶01 and P = 1.95×10^−7^; OR = 0.37; 95% CI, 0.24–0.55 for DPA1*01∶03-DPB1*04∶02). In the Korean subjects, a significant association of DPA1*01∶03-DPB1*04∶02 was also demonstrated; however, no association was observed for DPA1*01∶03-DPB1*04∶01. Because the observed number of each haplotype was small, none of the other haplotypes showed a significant association with protection against HBV infection.

In order to identify trans-ethnic DPA1-DPB1 haplotypes associated with HBV infection, a meta-analysis was performed. A meta-analysis further revealed that the DPA1*01∶03-DPB1*02∶01 haplotype was significantly associated with protection against HBV infection (P = 1.45×10^−5^; OR = 0.69; 95% CI, 0.58–0.82) (Fig. S1C).

## Discussion

Among 2.2 billion individuals worldwide who are infected with HBV, 15% of these are chronic carriers. Of chronic carriers, 10–15% develops LC, liver failure and HCC, and the remaining individuals eventually achieve a state of nonreplicative infection, resulting in HBsAg negative and anti-HBc positive, i.e. HBV-resolved individuals. To identify host genetic factors associated with HBV-related disease progression may lead HBV patients to discriminate individuals who need treatment.

The *HLA-DPA1* and *HLA-DPB1* genes were identified as host genetic factors significantly associated with CHB infection, mainly in Asian populations [7]–[12], and not in European populations [13]. In the previous association analyses of *HLA-DPB1* alleles with HBV infection, one risk allele HLA-DPB1*05∶01 (OR = 1.52; 95% CI, 1.31–1.76), and two protective alleles, HLA-DPB1*04∶01 (OR = 0.53; 95% CI, 0.34–0.80) and HLA-DPB1*04∶02 (OR = 0.47; 95% CI, 0.34–.64), were identified in the Japanese population [7]. In this study, we further identified a new risk allele HLA-DPB1*09∶01 (OR = 1.94; 95% CI, 1.45–2.62) for HBV infection and a new protective allele HLA-DPB1*02∶01 (OR = 0.71; 95% CI, 0.56–0.89) in the Japanese population, in addition to the previously reported alleles (Table S2) [7]. The discrepancy in the association of HLA-DPB1*09∶01 allele with risk for HBV infection in a previous study [7] results from the elevated frequency of HLA-DPB1*09∶01 in the controls (12.2%), which is higher than our controls (8.7%). In this study, healthy subjects were recruited as controls. In contrast, individuals that were registered in BioBank Japan as subjects with diseases other than CHB were recruited as controls in the previous study [7], which may have included patients with diseases with which HLA-DPB1*09∶01 is associated. Although no significant association of HLA-DPB1*09∶01 with risk for HBV infection was observed in the Korean subjects, HLA-DPB1*09∶01 appears to have a susceptible effect on HBV infection, as it showed the same direction of association. When the association analyses in Japanese and Korean subjects were combined in meta-analysis, the association was statistically significant (P = 1.36×10^−6^; OR = 1.97; 95% CI, 1.50–2.59). Thus, HLA-DPB1*09∶01 may be a Northeast Asian-specific allele associated with risk for HBV infection.

Moreover, a significant association of HLA-DPB1*13∶01 with risk of HBV infection (OR = 2.17; 95% CI, 1.40–3.47) was identified in the Thai subjects. However, the frequency of HLA-DPB1*13∶01 in Thai healthy controls (11.5% in the present study) reportedly varies, ranging from 15.4% to 29.5%, due to the population diversity [15]–[17]. Therefore, a replication analysis is required to confirm the association of HLA-DPB1*13∶01 with HBV infection in the Thai subjects. There were four other marginally associated *HLA-DPB1* alleles with low allele frequencies below 5% in HBV patients and healthy controls, including HLA-DPB1*28∶01, -DPB1*31∶01, -DPB1*100∶01, and -DPB1*105∶01, in the Hong Kong and Thai subjects. Because these infrequent alleles may have resulted from false positive associations, the association needs to be validated in a large number of subjects.

HLA-DPB1*02∶01 showed a significant association with protection against HBV infection in both Japanese and Hong Kong populations (Table S2); however, the HLA-DPB1*02∶01 allele was not associated with HBV infection in the previous study [7]. Although HLA-DPB1*02∶01 showed no association in either Korean or Thai populations, a significant association of HLA-DPB1*02∶01 with protection against HBV infection among four Asian populations was detected in meta-analysis (P = 5.22×10^−6^; OR = 0.68; 95% CI, 0.58–0.81) (Fig. S1B). We therefore conclude that the present finding is not a false positive.

A recent report showed that HLA-DPB1*02∶01∶02, *02∶02, *03∶01∶01, *04∶01∶01, *05∶01, *09∶01, and *14∶01 were significantly associated with response to booster HB vaccination in Taiwan neonatally vaccinated adolescents [18]. The HLA-DPB1*02∶01∶02, *02∶02, *03∶01∶01, *04∶01∶01, and *14∶01 were significantly more frequent in recipients whose post-booster titers of antibodies against HBV surface antigen (anti-HBs) were detectable, on the other hand, HLA-DPB1*05∶01 and *09∶01 were significantly more frequent in recipients who were undetectable. Moreover, the HLA-DPB1*05∶01 and *09∶01 significantly increase the likelihoods of undetectable pre-booster anti-HBs titers. These results seem consistent with our findings, in which HLA-DPB1*05∶01 and *09∶01 are associated with susceptibility to chronic hepatitis B infection.

We also identified a protective effect of HLA-DPB1*02∶01 allele on disease progression in Asian populations. Previous studies identified the association of HLA class II genes including *HLA-DQ* and *HLA-DR* with development of HBV related hepatocellular carcinoma in the Chinese population [6], [19], [20]. In this study using Japanese and Korean samples, we identified significant associations between HLA-DPB1*02∶01 and disease progression in CHB patients (P = 4.26×10^−5^; OR = 0.45; 95% CI, 0.30–0.67, for Japanese and P = 8.74×10^−4^; OR = 0.47; 95% CI, 0.29–0.75 for Korean) (Table S4). Although the association of HLA-DPB1*02∶01 with disease progression was weaker after adjustment for age and gender in Korean subjects (P = 2.54×10^−2^; OR = 0.55; 95% CI, 0.33–0.93), the same direction of association was observed (i.e. protective effect on disease progression) (Table 2). The protective effects of HLA-DPB1*02∶01 on disease progression showed a significant association after adjustment for age and gender in the Japanese population (P = 1.77×10^−4^; OR = 0.47; 95% CI, 0.32–0.70); moreover, a significant association between HLA-DPB1*02∶01 was observed among four Asian populations, under which population was adjusted by using dummy variables in a multivariate logistic regression analysis (P = 1.55×10^−7^; OR = 0.50; 95% CI, 0.39–0.65) (Table 2).

The *HLA-DPA1* and *HLA-DPB1* belong to the HLA class II alpha and beta chain paralogues, which make a heterodimer consisting of an alpha and a beta chain on the surface of antigen presenting cells. This HLA class II molecule plays a central role in the immune system by presenting peptides derived from extracellular proteins. We identified two susceptible haplotypes (DPA1*02∶02-DPB1*05∶01 and DPA1*02∶01-DPB1*09∶01) and three protective haplotypes (DPA1*01∶03-DPB1*04∶01, DPA1*01∶03-DPB1*04∶02, and HLA-DPA1*01∶03-DPB1*02∶01) to chronic hepatitis B infection, which may result in different binding affinities between HLA-DP subtypes and extracellular antigens. Although functional analyses of HLA-DP subtypes to identify HBV-related peptides are not fully completed, identification of susceptible and protective haplotypes as host genetic factors would lead us to understand the pathogenesis of HBV infection including viral factors.

In summary, we identified a new risk allele HLA-DPB1*09∶01, which was specifically observed in Northeast Asian populations, Japanese and Korean. Moreover, a new protective allele HLA-DPB1*02∶01 was identified among four Asian populations: Japanese, Korean, Hong Kong and Thai. The protective allele HLA-DPB1*02∶01 was associated with both chronic HBV infection and disease progression in chronic HBV patients. Identification of a total of five alleles, including two risk alleles (DPB1*09∶01 and DPB1*05∶01) and three protective alleles (DPB1*04∶01, DPB1*04∶02 and DPB1*02∶01), would enable HBV-infected individuals to be classified into groups according to the treatment requirements. Moreover, the risk and protective alleles for HBV infection and disease progression, identified in this study by means of trans-ethnic association analyses, would be key host factors to recognize HBV-derived antigen peptides. The present results may lead to subsequent functional studies into HLA-DP molecules and viral factors in order to understand the pathogenesis of HBV infection and development of hepatocellular carcinoma.

## Materials and Methods

### Ethics Statement

All study protocols conform to the relevant ethical guidelines, as reflected in the *a priori* approval by the ethics committee of National Center for Global Health and Medicine, and by the ethics committees of all participating universities and hospitals, including The University of Tokyo, Japanese Red Cross Kanto-Koshinetsu Block Blood Center, The University of Hong Kong, Chulalongkorn University, Yonsei University College of Medicine, Nagoya City University Graduate School of Medical Sciences, Musashino Red Cross Hospital, Tokyo Medical and Dental University, Teine Keijinkai Hospital, Hokkaido University Graduate School of Medicine, Kurume University School of Medicine, Okayama University Graduate School of Medicine, Yamaguchi University Graduate School of Medicine, Tottori University, Kyoto Prefectural University of Medicine, Osaka City University Graduate School of Medicine, Nagoya Daini Red Cross Hospital, Ehime University Graduate School of Medicine, Kanazawa University Graduate School of Medicine, National Hospital Organization Osaka National Hospital, Iwate Medical University, Kawasaki Medical College, Shinshu University School of Medicine, Saitama Medical University, Kitasato University School of Medicine, Saga Medical School, and University of Tsukuba.

Written informed consent was obtained from each patient who participated in this study and all samples were anonymized. For Japanese healthy controls, 419 individuals were de-identified with information about gender, and all were recruited after obtaining verbal informed consent in Tokyo prior to 1990. For the 419 Japanese healthy individuals, written informed consent was not obtained because the blood sampling was conducted before the “Ethical Guidelines for Human Genome and Genetic Sequencing Research” were established in Japan. Under the condition that DNA sample is permanently de-linked from the individual, this study was approved by the Research Ethics Committee of National Center for Global Health and Medicine.

### Characteristics of studied subjects

All of the 3,167 genomic DNA samples were collected from individuals with HBV, HBV-resolved individuals (HBsAg-negative and anti-HBc-positive) and healthy controls at 26 multi-center hospitals throughout Japan, Korea, Hong Kong, and Thailand (Table 1). In a total of 1,291 Japanese and 586 Korean samples, 1,191 Japanese individuals and all 586 Korean individuals were included in our previous study [9]. With regard to additional Japanese individuals, we collected samples from 48 healthy controls at Kohnodai Hospital, and 52 HBV patients at Okayama University Hospital and Ehime University Hospital, including 26 individuals with LC and 26 individuals with HCC. A total of 661 Hong Kong samples and 629 Thai samples were collected at Queen Mary Hospital and Chulalongkorn University, respectively.

HBV status was measured based on serological results for HBsAg and anti-HBc with a fully automated chemiluminescent enzyme immunoassay system (Abbott ARCHITECT; Abbott Japan, Tokyo, Japan, or LUMIPULSE f or G1200; Fujirebio, Inc., Tokyo, Japan). For clinical staging, inactive carrier (IC) state was defined by the presence of HBsAg with normal ALT levels over 1 year (examined at least four times at 3-month intervals) and without evidence of liver cirrhosis. Chronic hepatitis (CH) was defined by elevated ALT levels (>1.5 times the upper limit of normal [35 IU/L]) persisting over 6 months (by at least 3 bimonthly tests). Acute exacerbation (AE) of chronic hepatitis B was defined as an elevation of ALT to more than 10 times the upper limit of normal (ULN, 58 IU/L) and bilirubin to at least three times ULN (15 µmol/L). LC was diagnosed principally by ultrasonography (coarse liver architecture, nodular liver surface, blunt liver edges and hypersplenism), platelet counts<100,000/cm^3^, or a combination thereof. Histological confirmation by fine-needle biopsy of the liver was performed as required. HCC was diagnosed by ultrasonography, computerized tomography, magnetic resonance imaging, angiography, tumor biopsy or a combination thereof.

The Japanese control samples from HBV-resolved subjects (HBsAg-negative and anti-HBc-positive) at Nagoya City University-affiliated healthcare center were used by comprehensive agreement (anonymization in a de-identified manner) in this study. Some of the unrelated and anonymized Japanese healthy controls were purchased from the Japan Health Science Research Resources Bank (Osaka, Japan). One microgram of purified genomic DNA was dissolved in 100 µl of TE buffer (pH 8.0) (Wako, Osaka, Japan), followed by storage at −20°C until use.

### Genotyping of *HLA-DPA1* and *HLA-DPB1* alleles

High resolution (4-digit) genotyping of *HLA-DPA1* and *-DPB1* alleles was performed for HBV patients, resolved individuals, and healthy controls in Japan, Korea, Hong Kong, and Thailand. LABType SSO HLA DPA1/DPB1 kit (One Lambda, CA) and a Luminex Multi-Analyte Profiling system (xMAP; Luminex, Austin, TX) were used for genotyping, in according with the manufacturer's protocol. Because of the small quantity of genomic DNA in some Korean samples, we performed whole genome amplification for a total of 486 samples using GenomiPhi v2 DNA Amplification kit (GE Healthcare Life Sciences, UK), in accordance with the manufacturer's instruction.

A total of 2,895 samples were successfully genotyped and characteristics of these samples are summarized in Table S1.

### Statistical analysis

Fisher's exact test in two-by-two cross tables was used to examine the associations between *HLA-DP* allele and chronic HBV infection or disease progression in chronic HBV patients, using statistical software R2.9. To avoid false-positive results due to multiple testing, significance levels were adjusted based on the number of observed alleles at each locus in each population. For *HLA-DPA1* alleles, the number of observed alleles was 3 in Japanese, 4 in Korean, 5 in Hong Kong, and 5 in Thai subjects. Therefore, the significant levels for α were set at α = 0.05/3 in Japanese, α = 0.05/4 in Korean, α = 0.05/5 in Hong Kong, and α = 0.05/5 in Thai subjects. In the same way, significant levels for *HLA-DPB1* alleles were α = 0.05/10, 0.05/11, 0.05/12, and 0.05/16, respectively. Multivariate logistic regression analysis adjusted for age and sex (used as independent variables) was applied to assess associations between the number of DPB1*02∶01 alleles (i.e., 0, 1, or 2) and disease progression in CHB patients. To examine the effect of DPB1*02∶01 allele on disease progression in all populations, population was further adjusted by using three dummy variables (i.e., (c1, c2, c3) = (0, 0, 0) for Japanese, (1, 0, 0) for Korean, (0, 1, 0) for Hong Kong, and (0, 0, 1) for Thai) in a multivariate logistic regression analysis. We obtained the following regression equation: logit(p) = −3.905+0.083*age+(−0.929)*sex+(−0.684)*DPB1*02∶01+1.814*c1+(−0.478)*c2+0.782*c3. Significance levels in the analysis of disease progression in CHB patients were set as α =  0.05/10 in Japanese, α = 0.05/11 in Korean, α = 0.05/15 in Hong Kong, and α = 0.05/15 in Thai subjects. The phase of each individual (i.e., a combination of two *DPA1-DPB1* haplotypes) was estimated using PHASE software [21], assuming samples are selected randomly from a general population. In comparison of the estimated *DPA1-DPB1* haplotype frequencies, significant levels were set as α = 0.05/14 in Japanese, α = 0.05/17 in Korean, α = 0.05/17 in Hong Kong, and α = 0.05/18 in Thai subjects. Meta-analysis was performed using the DerSimonian-Laird method (random-effects model) in order to calculate pooled OR and its 95% confidence interval (95% CI). We applied meta-analysis for alleles with frequency>1% in all four Asian populations. The significance levels in meta-analysis were adjusted by the total number of statistical tests; α = 0.05/20 for *DPA1* alleles, α = 0.05/57 for *DPB1* alleles, and α = 0.05/74 for *DPA1-DPB1* haplotypes.

## Supporting Information

Figure S1 **Comparison of odds ratios in association analyses for** ***HLA-DP*** **with chronic HBV infection among four Asian populations: (A)** ***HLA-DPA1*** **alleles; (B)** ***HLA-DPB1*** **alleles; and (C)** ***HLA DPA1-DPB1*** **haplotypes. Meta-analysis was performed using the DerSimonian-Laird method (random-effects model) to calculate pooled OR and its 95% confidence interval (95% CI).** Bold depicts a statistically significant association after correction of significance level.
(DOCX)

Table S1 **Individuals with successfully genotyped for** ***HLA-DPA1*** **and** ***HLA-DPB1*** **.**
(DOCX)

Table S2 **Frequencies of HLA-DP alleles in HBV patients and healthy controls among Asian populations.**
(XLSX)

Table S3 **Frequencies of HLA-DP alleles in HBV patients and resolved individuals among Asian populations.**
(XLSX)

Table S4 **Associations of HLA-DPB1 alleles with disease progression in CHB patients among Asian populations.**
(XLSX)

Table S5 **Estimated frequencies of HLA DPA1-DPB1 haplotypes in HBV patients and healthy controls among Asian populations.**
(XLSX)

## References

[1] Chen DS (1993) From hepatitis to hepatoma: lessons from type B viral hepatitis. Science 262: 369–370. 8211155 10.1126/science.8211155

[2] Custer B, Sullivan SD, Hazlet TK, Iloeje U, Veenstra DL, et al (2004) Global epidemiology of hepatitis B virus. J Clin Gastroenterol 38: S158–168. 15602165 10.1097/00004836-200411003-00008

[3] Zidan A, Scheuerlein H, Schule S, Settmacher U, Rauchfuss F (2012) Epidemiological pattern of hepatitis B and hepatitis C as etiological agents for hepatocellular carcinoma in iran and worldwide. Hepat Mon 12: e6894. 23233864 10.5812/hepatmon.6894 PMC3517809

[4] Pungpapong S, Kim WR, Poterucha JJ (2007) Natural history of hepatitis B virus infection: an update for clinicians. Mayo Clin Proc 82: 967–975. 17673066 10.4065/82.8.967

[5] Kim DW, Lee SA, Hwang ES, Kook YH, Kim BJ (2012) Naturally occurring precore/core region mutations of hepatitis B virus genotype C related to hepatocellular carcinoma. PLoS One 7: e47372. 23071796 10.1371/journal.pone.0047372 PMC3468518

[6] Jiang DK, Sun J, Cao G, Liu Y, Lin D, et al (2013) Genetic variants in STAT4 and HLA-DQ genes confer risk of hepatitis B virus-related hepatocellular carcinoma. Nat Genet 45: 72–75. 23242368 10.1038/ng.2483 PMC4105840

[7] Kamatani Y, Wattanapokayakit S, Ochi H, Kawaguchi T, Takahashi A, et al (2009) A genome-wide association study identifies variants in the HLA-DP locus associated with chronic hepatitis B in Asians. Nat Genet 41: 591–595. 19349983 10.1038/ng.348

[8] Mbarek H, Ochi H, Urabe Y, Kumar V, Kubo M, et al (2011) A genome-wide association study of chronic hepatitis B identified novel risk locus in a Japanese population. Hum Mol Genet 20: 3884–3892. 21750111 10.1093/hmg/ddr301

[9] Nishida N, Sawai H, Matsuura K, Sugiyama M, Ahn SH, et al (2012) Genome-wide association study confirming association of HLA-DP with protection against chronic hepatitis B and viral clearance in Japanese and Korean. PLoS One 7: e39175. 22737229 10.1371/journal.pone.0039175 PMC3380898

[10] Guo X, Zhang Y, Li J, Ma J, Wei Z, et al (2011) Strong influence of human leukocyte antigen (HLA)-DP gene variants on development of persistent chronic hepatitis B virus carriers in the Han Chinese population. Hepatology 53: 422–428. 21274863 10.1002/hep.24048 PMC3056070

[11] An P, Winkler C, Guan L, O'Brien SJ, Zeng Z (2011) A common HLA-DPA1 variant is a major determinant of hepatitis B virus clearance in Han Chinese. J Infect Dis 203: 943–947. 21402545 10.1093/infdis/jiq154 PMC3068033

[12] Li J, Yang D, He Y, Wang M, Wen Z, et al (2011) Associations of HLA-DP variants with hepatitis B virus infection in southern and northern Han Chinese populations: a multicenter case-control study. PLoS One 6: e24221. 21904616 10.1371/journal.pone.0024221 PMC3164164

[13] Vermehren J, Lotsch J, Susser S, Wicker S, Berger A, et al (2012) A common HLA-DPA1 variant is associated with hepatitis B virus infection but fails to distinguish active from inactive Caucasian carriers. PLoS One 7: e32605. 22448225 10.1371/journal.pone.0032605 PMC3308944

[14] Sawai H, Nishida N, Mbarek H, Matsuda K, Mawatari Y, et al (2012) No association for Chinese HBV-related hepatocellular carcinoma susceptibility SNP in other East Asian populations. BMC Med Genet 13: 47. 22712471 10.1186/1471-2350-13-47 PMC3407509

[15] Chandanayingyong D, Stephens HA, Fan L, Sirikong M, Longta P, et al (1994) HLA-DPB1 polymorphism in the Thais of Southeast Asia. Hum Immunol 40: 20–24. 8045789 10.1016/0198-8859(94)90017-5

[16] Chandanayingyong D, Stephens HA, Klaythong R, Sirikong M, Udee S, et al (1997) HLA-A, -B, -DRB1, -DQA1, and -DQB1 polymorphism in Thais. Hum Immunol 53: 174–182. 9129976 10.1016/S0198-8859(96)00284-4

[17] Maneemaroj R, Stephens HA, Chandanayingyong D, Longta K, Bejrachandra S (1997) HLA class II allele frequencies in northern Thais (Kamphaeng Phet). J Med Assoc Thai 80 Suppl 1 S20–24. 9347641

[18] Wu TW, Chu CC, Ho TY, Chang Liao HW, Lin SK, et al.. (2013) Responses to booster hepatitis B vaccination are significantly correlated with genotypes of human leukocyte antigen (HLA)-DPB1 in neonatally vaccinated adolescents. Hum Genet. 10.1007/s00439-013-1320-5 23739870

[19] Hu L, Zhai X, Liu J, Chu M, Pan S, et al (2012) Genetic variants in human leukocyte antigen/DP-DQ influence both hepatitis B virus clearance and hepatocellular carcinoma development. Hepatology 55: 1426–1431. 22105689 10.1002/hep.24799

[20] Li S, Qian J, Yang Y, Zhao W, Dai J, et al (2012) GWAS identifies novel susceptibility loci on 6p21.32 and 21q21.3 for hepatocellular carcinoma in chronic hepatitis B virus carriers. PLoS Genet 8: e1002791. 22807686 10.1371/journal.pgen.1002791 PMC3395595

[21] Stephens M, Smith NJ, Donnelly P (2001) A new statistical method for haplotype reconstruction from population data. Am J Hum Genet 68: 978–989. 11254454 10.1086/319501 PMC1275651

