# Supplementary material for: New Susceptibility and Resistance HLA-DP Alleles to HBV-Related Diseases Identified by a Trans-Ethnic Association Study in Asia
Source: PLoS One. 2014 Feb 10;9(2):e86449. doi: 10.1371/journal.pone.0086449 (PMC3919706; doi:10.1371/journal.pone.0086449)

Figure S1. Comparison of odds ratios in association analyses for HLA-DP with chronic HBV infection among four Asian populations


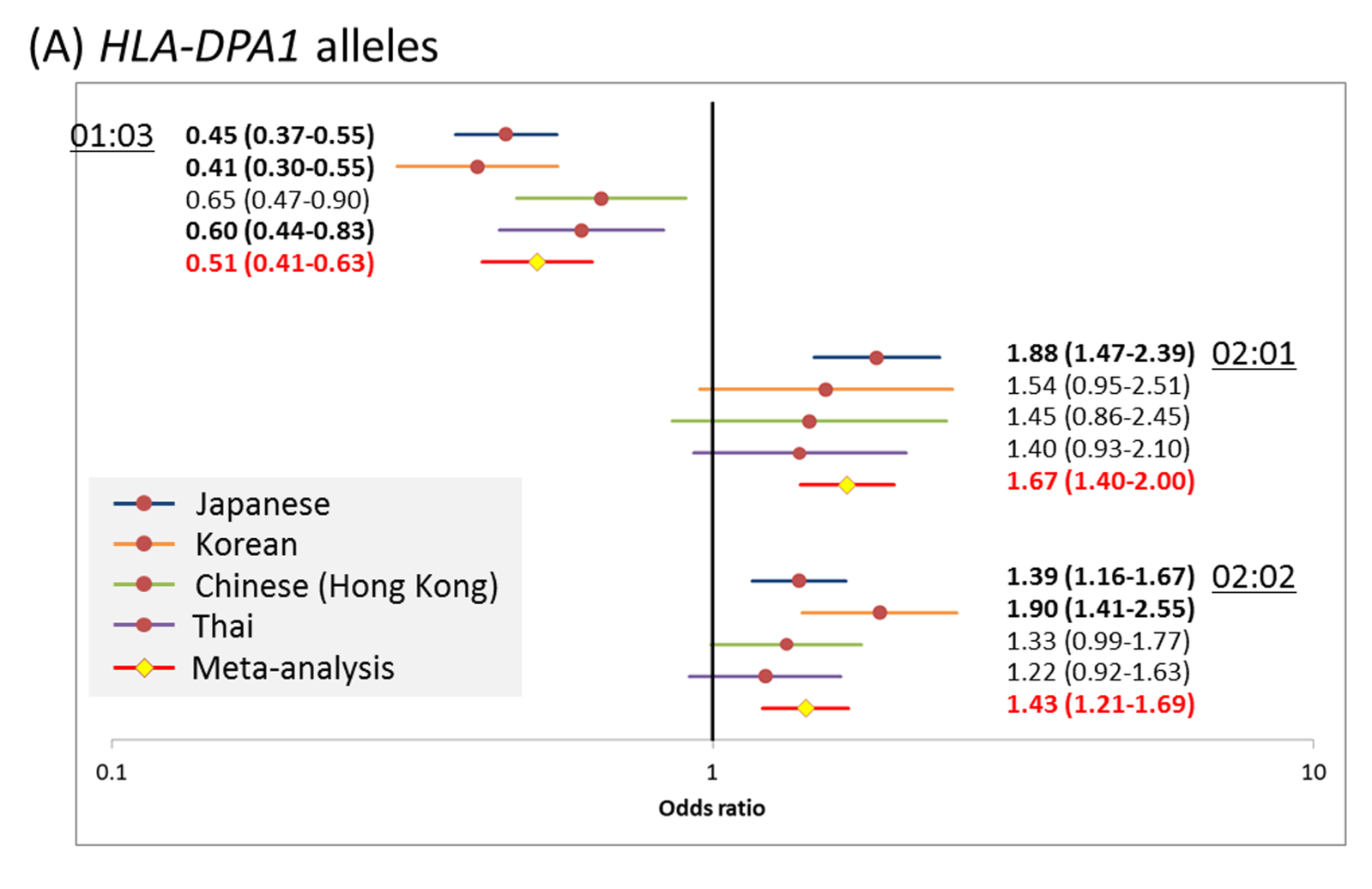


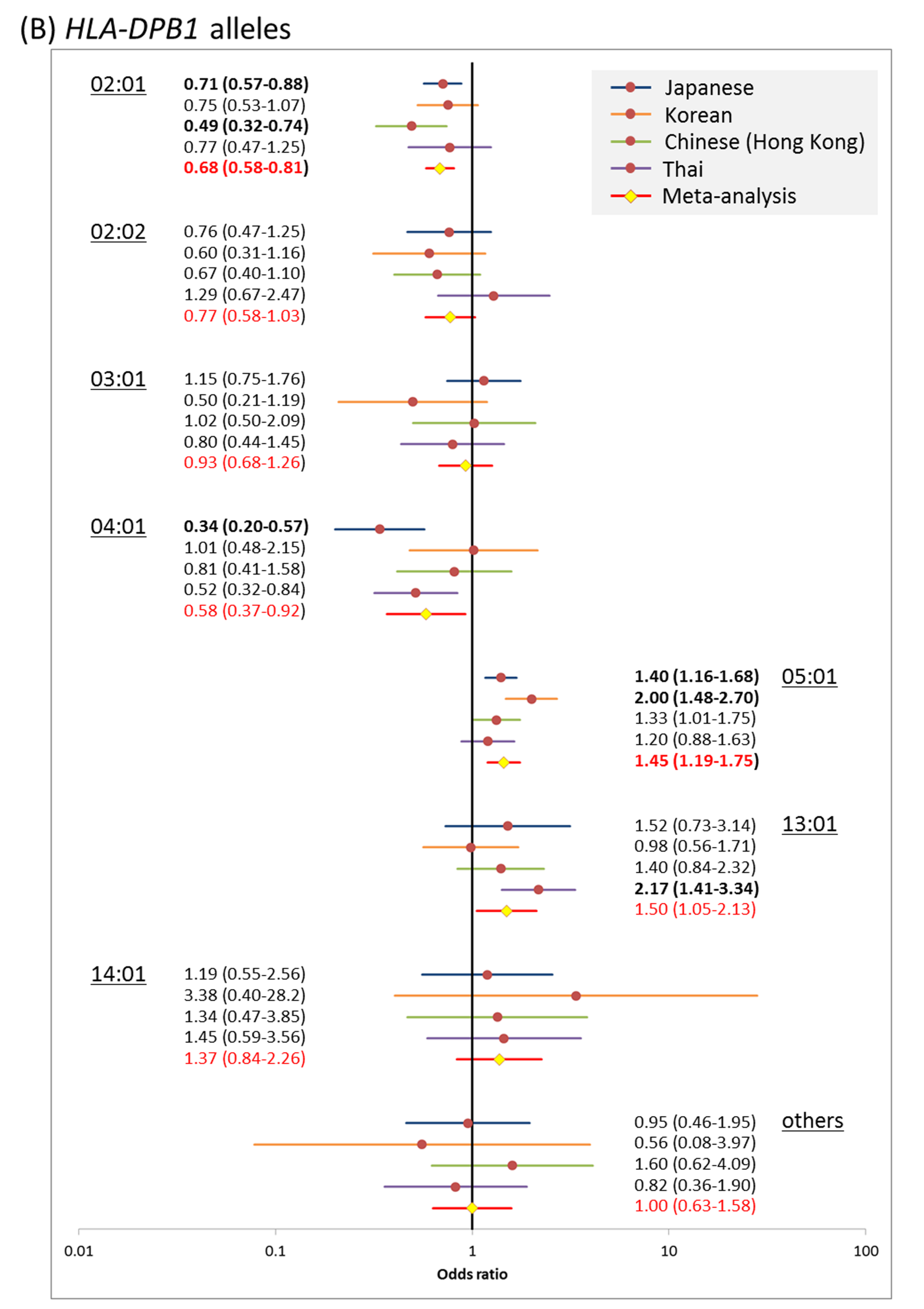


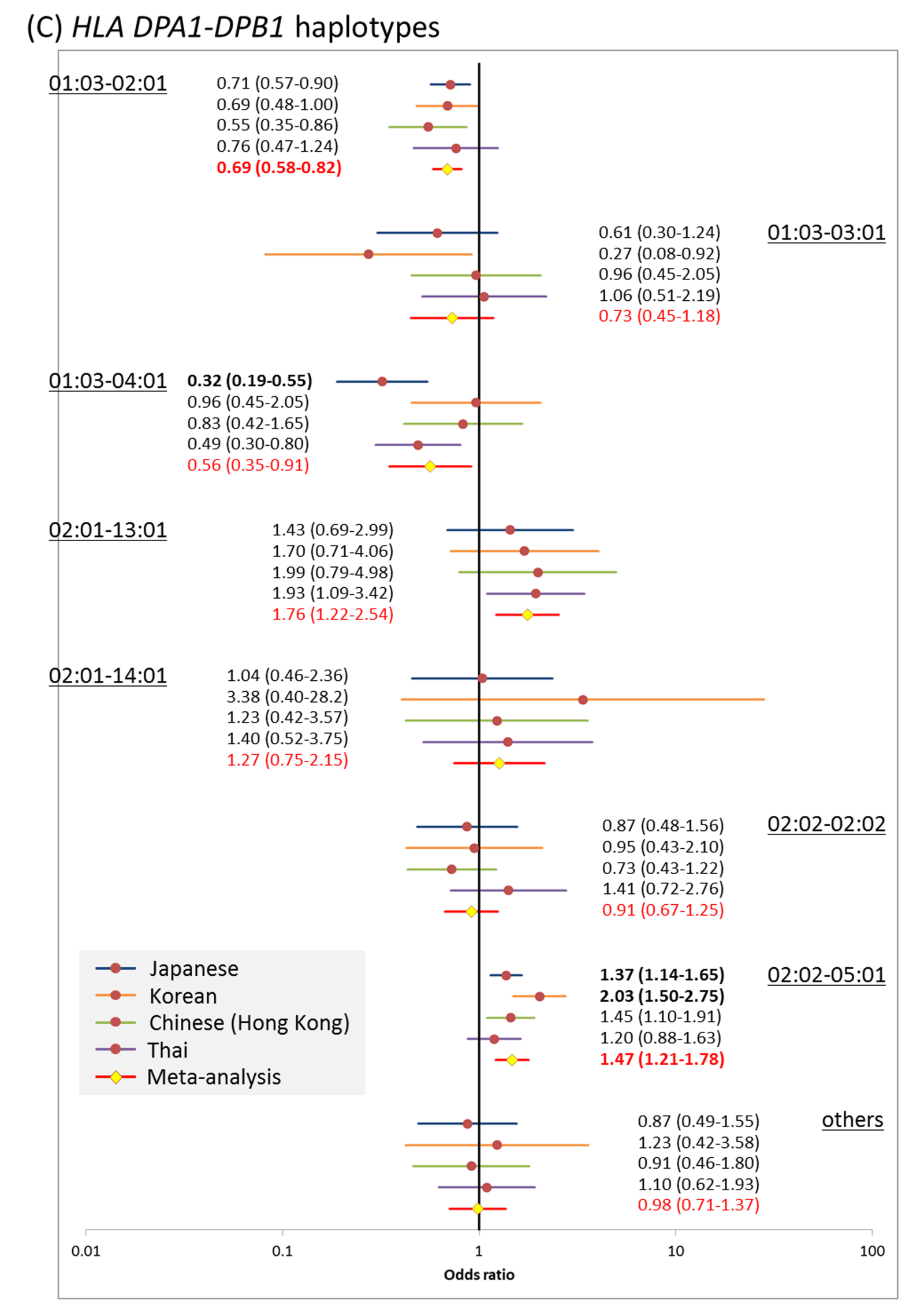

Supplement: Figure S1 — Comparison of odds ratios in association analyses for HLA-DP with chronic HBV infection among four Asian populations: (A) HLA-DPA1 alleles; (B) HLA-DPB1 alleles; and (C) HLA DPA1-DPB1 haplotypes. Meta-analysis was performed using the DerSimonian-Laird method (random-effects model) to calculate pooled OR and its 95% confidence interval (95% CI). Bold depicts a statistically significant association after correction of significance level. (DOCX) [file pone.0086449.s001.docx]
