# Supplementary material for: New Susceptibility and Resistance HLA-DP Alleles to HBV-Related Diseases Identified by a Trans-Ethnic Association Study in Asia
Source: PLoS One. 2014 Feb 10;9(2):e86449. doi: 10.1371/journal.pone.0086449 (PMC3919706; doi:10.1371/journal.pone.0086449)
Supplement: Table S1 — Individuals with successfully genotyped for HLA-DPA1 and HLA-DPB1 . (DOCX) [file pone.0086449.s002.docx]

Table S1. Individuals with successfully genotyped for *HLA-DPA1* and *HLA-DPB1*

|  |  |  |  |  |  |
| --- | --- | --- | --- | --- | --- |
| Population | | Japanese | Korean | Hong Kong | Thai |
|  |  |  |  |  |  |
|  |  |  |  |  |  |
| HBV patients | Total number of samples | 489 | 340 | 281 | 390 |
|  | Successfully genotyped | 488 | 251 | 280 | 369 |
|  | IC | 114 | - | - | - |
|  | CH | 147 | 108 | 186 | 194 |
|  | AE | 21 | - | - | - |
|  | LC | 37 | - | - | - |
|  | HCC | 169 | 143 | 94 | 175 |
|  | Mean age (y)  (min-max) | 57.2 (20-84) | 46.4 (18-74) | 57.9 (32-86) | 51.9 (21-84) |
|  | Gender (M/F) | 337/151 | 194/57 | 238/42 | 272/97 |
|  |  |  |  |  |  |
|  |  |  |  |  |  |
| Resolved individuals* | Total number of samples | 335 | 106 | 190 | 113 |
|  | Successfully genotyped | 326 | 106 | 84 | 109 |
|  | HCV (-) | 241 | 106 | 84 | 109 |
|  | HCV (+) | 85 | - | - | - |
|  | Mean age (y) (min-max) | 59.8 (18-87) | 43.1 (12-66) | 39.6 (18-59) | 48.3 (39-66) |
|  | Gender (M/F) | 170/156 | 61/45 | 57/27 | 82/27 |
|  |  |  |  |  |  |
|  |  |  |  |  |  |
| Healthy controls | Total number of samples | 467 | 140 | 190 | 126 |
|  | Successfully genotyped | 464 | 140 | 156 | 122 |
|  | Mean age (y)  (min-max) | 39.1** (23-64) | 33.7 (1-59) | 25.5 (16-58) | 46.6 (38-79) |
|  | Gender (M/F) | 367/97 | 67/73 | 81/75 | 70/52 |
|  |  |  |  |  |  |

Abbreviation: IC, Inactive Carrier; CH, Chronic Hepatitis; AE, Acute Exacerbation; LC, Liver Cirrhosis; HCC, Hepatocellular Carcinoma

* Resolved individuals were HBsAg negative and HBcAb positive.

** 419 of 464 healthy controls were de-identified without information on age.
